# The Accuracy of Patient-Specific Instrumentation with Laser Guidance in a Dynamic Total Hip Arthroplasty: A Radiological Evaluation

**DOI:** 10.3390/s21124232

**Published:** 2021-06-20

**Authors:** Andrea Ferretti, Ferdinando Iannotti, Lorenzo Proietti, Carlo Massafra, Attilio Speranza, Andrea Laghi, Raffaele Iorio

**Affiliations:** 1 Orthopaedic and Traumatology Unit, Sant’Andrea Hospital, “Sapienza” University of Rome, 00189 Rome, Italy; andrea.ferretti@uniroma1.it (A.F.); lorenzo.proietti@uniroma1.it (L.P.); carlo.massafra@uniroma1.it (C.M.); arsperanza@gmail.com (A.S.); raffaele.iorio@uniroma1.it (R.I.); 2 Department of Radiology, Sant’Andrea Hospital, “Sapienza” University of Rome, 00189 Rome, Italy; andrea.laghi@uniroma1.it

**Keywords:** PSI, THA, patient-specific dynamic planning, spinopelvic kinematic, pelvic tilt, functional orientation of components

## Abstract

The functional positioning of components in a total hip arthroplasty (THA) and its relationship with individual lumbopelvic kinematics and a patient’s anatomy are being extensively studied. Patient-specific kinematic planning could be a game-changer; however, it should be accurately delivered intraoperatively. The main purpose of this study was to verify the reliability and accuracy of a patient-specific instrumentation (PSI) and laser-guided technique to replicate preoperative dynamic planning. Thirty-six patients were prospectively enrolled and received dynamic hip preoperative planning based on three functional lateral spinopelvic X-rays and a low dose CT scan. Three-dimensional (3D) printed PSI guides and laser-guided instrumentation were used intraoperatively. The orientation of the components, osteotomy level and change in hip length and offset were measured on postoperative CT scans and compared with the planned preoperative values. The length of surgery was compared with that of a matched group of thirty-six patients who underwent a conventional THA. The mean absolute deviation from the planned inclination and anteversion was 3.9° and 4.4°, respectively. In 92% of cases, both the inclination and anteversion were within +/− 10° of the planned values. Regarding the osteotomy level, offset change and limb length change, the mean deviation was, respectively, 1.6 mm, 2.6 mm and 2 mm. No statistically significant difference was detected when comparing the planned values with the achieved values. The mean surgical time was 71.4 min in the PSI group and 60.4 min in the conventional THA group (*p* < 0.05). Patient-specific and laser-guided instrumentation is safe and accurately reproduces dynamic planning in terms of the orientation of the components, osteotomy level, leg length and offset. Moreover, the increase in surgical time is negligible.

## 1. Introduction

The proper positioning of components in a total hip arthroplasty (THA) represents a crucial aspect for assuring an adequate stability, equalizing limb length discrepancies and recreating the appropriate offset. Accurate alignment is key to optimizing the functional outcomes and to reducing the rates of dislocation, impingement, aseptic loosening and other wear-related complications [1,2].

The orientation of the acetabular cup is one of the most important factors under the surgeon’s control. Malposition of the acetabular cup could lead to instability, edge loading, osteolysis and squeezing, having a significant effect on the implant performance and patient outcomes [3,4].

In 1978, Lewinnek et al. [5] suggested a relatively safe range of orientation for the cup; 40° ± 10° of inclination and 15° ± 10° of anteversion (safe zone). However, a few authors have demonstrated that up to 60% of dislocations occur in implants positioned within the safe zone [6]. This has led to suggestions that the idea of one generic safe zone may be misleading [7] especially taking into account that these alignment guidelines have been evaluated on radiographs in a supine, static position [8]. However, the acetabular orientation is not a static parameter; it is related to the pelvic movement in the sagittal plane that changes the acetabular functional version and inclination during activities of daily living [8]. It has been reported that the pelvis normally rotates posteriorly moving between supine, standing and sitting positions thus increasing the anterior opening of the acetabulum [9,10,11].

As a consequence, a component placed within the safe zone intraoperatively could be unsafe and inappropriate when that patient is walking, sitting or standing [12]. As a matter of fact, edge loading, impingement and dislocation occur more commonly during activities rather than in a static position [8]. Moreover, sagittal postural balance varies from patient to patient and it is further influenced by multiple factors including degenerative disk disease or lumbar fusion [13]. Patients with a stiff spine have an increased risk of instability after a THA as a result of altered spinopelvic kinematics [14,15].

This scenario emphasizes the importance of the highly patient-specific placement of components based upon an individual preoperative functional assessment. Several studies recommended performing standing and sitting lateral radiographs to evaluate for a spinopelvic imbalance [11,12,13,16]. Pierrepont et al. recommend using the flexed-seated position to evaluate the patient’s functional pelvic tilt in flexion as posterior edge loading and dislocation occur more commonly when the patient is rising from a chair or bending; in this posture, the flexed torso forces the pelvis to tilt anteriorly [8].

On the femoral side, due to the complex morphology and variability of the proximal medullary canal, the level and angle of the femoral neck osteotomy can influence the leg length and stem anteversion especially in cementless implants. A femoral osteotomy and stem placement should be performed to optimize the leg length discrepancy and to reproduce the femoral offset [3,17,18].

A three-dimensional (3D) reconstruction of a patient’s anatomy along with functional planning can help to better reproduce the optimization of the femoral anatomy and, at the same time, the mating of the femoral head in the cup throughout all body positions, thus lowering the risk of impingement [19].

Patient-specific kinematic planning is a key factor; however, it is essential to accurately deliver it intraoperatively. A mismatch has been reported between preoperative planning and the final positioning of components ranging from 20% to 40% of the cases [20,21,22].

The Optimized Positioning System™ (OPS™, Corin Ltd., Cirencester, UK) is a new system that provides preoperative planning with a patient-specific component alignment as a result of a patient dynamic analysis. Furthermore, patient-specific instrumentation (PSI) with laser guidance is provided to deliver the targeted component alignment.

The computer-based preoperative planning aims to optimize the position and the functional orientation of the implant with the purpose of avoiding a potential impingement and instability during a patient’s daily life activities. The functional analysis is based on a comprehensive imaging study, which includes low dose CT scans and lateral X-rays in three functional positions: standing, step-up and flexed-seated. Based on the acquired images and preoperative planning, a set of 3D printed patient-specific femoral cutting and acetabular reaming guides is then provided to intraoperatively reproduce the planned cup and stem alignment.

The main purpose of this study was to verify the reliability and accuracy of the OPS^TM^ PSI guides and laser-guided technique to replicate preoperative dynamic planning regarding the cup orientation, osteotomy level and change in limb length and offset. The secondary objective of this study was to determine the impact of this delivery system in the operating time compared with a conventional THA.

## 2. Materials and Methods

All patients with a diagnosis of hip osteoarthritis requiring a THA were eligible for inclusion during a six-month period beginning in January 2019. The exclusion criteria applied were as follows: previous hip surgery, hip ankylosis, contralateral hip prosthesis and the disability to sit and stand during the radiographic study.

All patients who agreed to follow the preoperative and postoperative imaging protocol were enrolled in this prospective study. Two patients were excluded as they were unable to preoperatively undergo an X-ray in the flexed-seated position due to severe hip stiffness.

Thirty-six consecutive patients were included and received OPS™ (Corin Ltd., Cirencester, UK) dynamic hip preoperative planning. An anteroposterior radiograph of the pelvis, three functional lateral spinopelvic X-rays (standing, flexed-seated and stepping-up) and a low dose CT scan were performed preoperatively. All of the images were sent to the manufacturer for the analysis.

**Figure 1:**
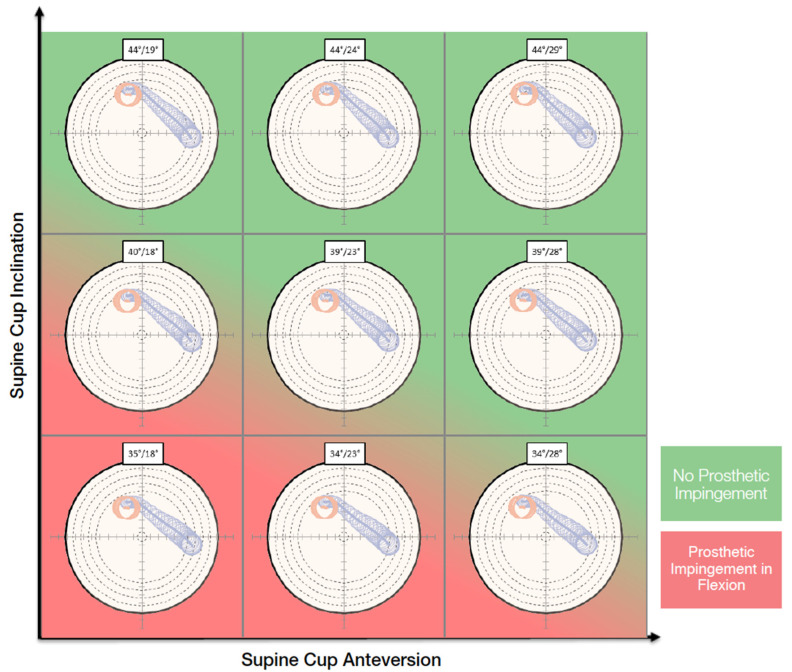
Nine different cup orientations are provided to the surgeon with an indication of the risk of impingement.

On the functional images, the pelvic tilt with pelvic rotation from different positions, the pelvic incidence, the lumbar lordotic angle and the lumbar flexion were measured. The kinematic inputs drove dynamic planning. The purpose of the OPS™ is to avoid the risk of edge loading by optimizing the cup and stem orientation according to the patient’s spinopelvic mobility and anatomy. The surgeon can choose an optimal cup orientation for the patient evaluating contact patch paths that are presented in polar plots for nine different cup orientations (Figure 1). The system also aims to assure an adequate range of motion by reducing the risk of impingement and to optimize the leg length and offset to restore the native femoral head center.

The preoperative plan with the virtual implant templating was approved before the operation by the surgeon. The pre-plan included a proposed cup type, size and orientation; the stated cup alignment was referenced to the coronal plane when the subject was supine. Planning also included the level of the osteotomy, stem type, position and size and estimated change in the leg length and offset compared with the preoperative state. All of these parameters were recorded to make a comparison with the corresponding postoperative measurements. After planning approval, the acetabular and femoral 3D printed PSI guides were delivered.

This study includes the first 36 cases performed with OPS™ by the same experienced surgeon.

### 2.1. Surgical Technique

**Figure 2:**
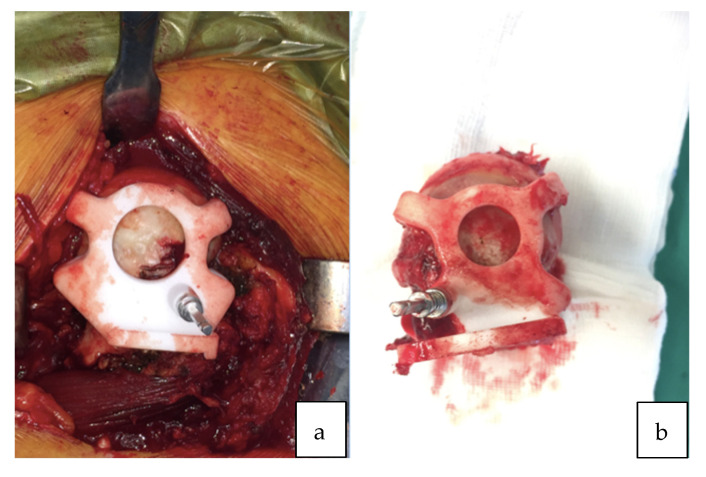
(**a**) Patient-specific femoral guide fitted to the proximal femur. (**b**) Femoral guide fitted to the proximal femur after the osteotomy cut.

**Figure 3:**
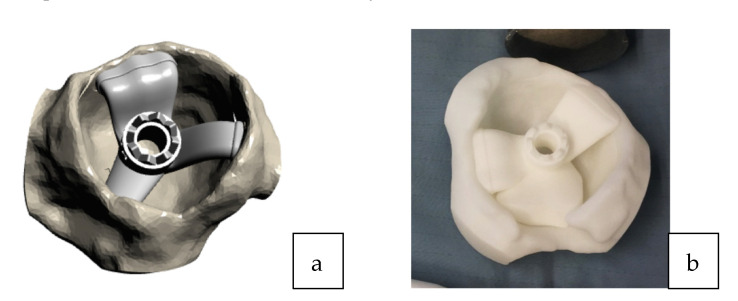
(**a**) 3D acetabular model with the patient-specific guide in place. (**b**) The 3D printed form of the patient’s acetabulum is used as a model by the surgeon to guide them in the positioning of the laser guide.

**Figure 4:**
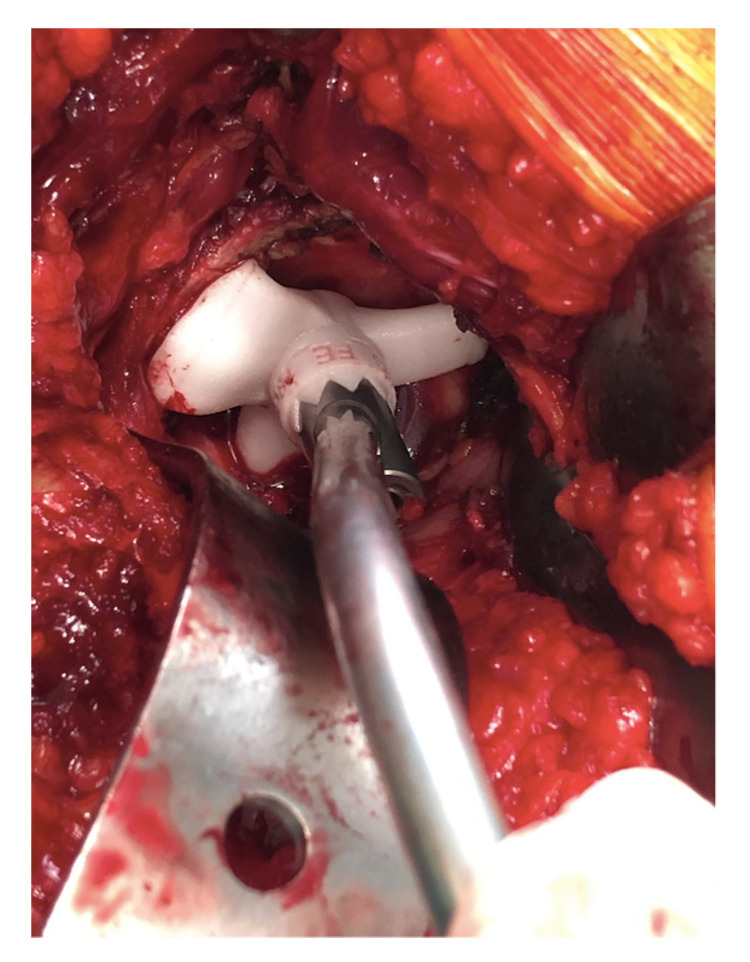
Acetabular guide adapted inside the acetabulum. Once the guide is positioned, the curved handle is attached with a laser that will indicate the reference point for the positioning of the acetabular component.

**Figure 5:**
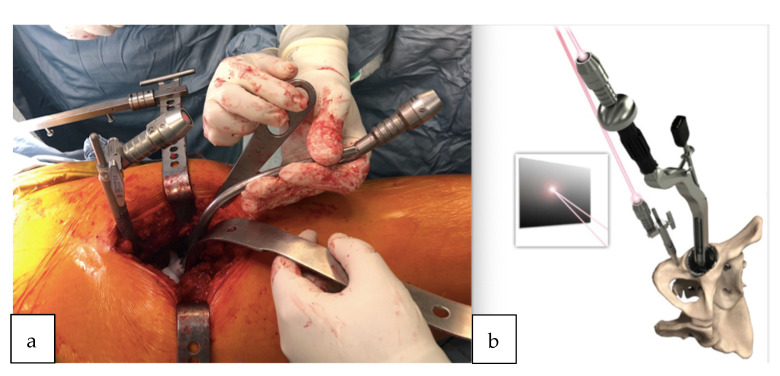
(**a**) Pelvic reference pin and acetabular guide introducer topped with the laser pointer. The lights of both lasers were made to converge on the wall of the theater. (**b**) Demonstration of the removable laser adapted also to the top of the impaction handle; the coincidence of the lights is checked again to guide the cup impaction.

All surgeries were performed using a direct lateral surgical approach in a lateral decubitus position. The femoral exposure was performed as a standard practice. The femoral PSI guide was fitted on the femur and it was used to perform the planned osteotomy cut (Figure 2a,b). The acetabulum was then exposed and the soft tissue was removed as needed to fit the acetabular PSI guide (Figure 3a,b). A laser was attached to the guide using a curved handle (Figure 4). A reference pin topped with an articulated laser pointer was placed on the superior edge of the acetabulum. The light of the second laser was made to converge on the light of the first laser at the theater wall. (Figure 5a,b). The acetabular PSI and the corresponding laser were removed. A new laser was adapted to the top of the acetabular reamer. The reaming was guided by assuring the coincidence of the projected lights (reference laser and acetabular reamer laser) in all phases of the procedure. Afterwards, a laser was also adapted to the top of the impaction handle and the coincidence of the lights was checked again to guide the cup impaction (Figure 5b). After positioning the cup, the reference pin was removed.

Femoral broaching was then performed according to the preoperative plan; an image showing a section in the transverse plane of the femoral template at the level of the osteotomy was used as a reference to obtain the planned stem anteversion. The final implant of the stem was performed using the conventional method. The range of motion, impingement and stability were then tested in the standard way.

All patients received an uncemented acetabular cup (Trinity™ cup, Corin Ltd., Cirencester, UK) and a taper wedged blade stem (TriFit TS™, Corin Ltd., Cirencester, UK).

### 2.2. Postoperative CT Evaluation

**Figure 6:**
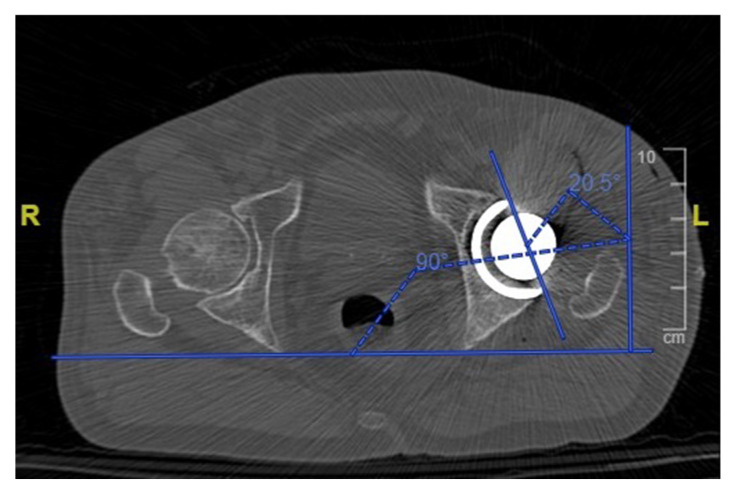
Measurement of the acetabular cup anteversion angle on a postoperative CT scan.

One month postoperatively, all patients received a low dose CT scan of the pelvis and femur with a mean dose of 2.8 to 4.0 mSv per scan. The inclination and anteversion of the cup (Figure 6) were accurately measured for each patient and compared with the planned preoperative values. The height of the femoral neck resection was measured with reference to the lesser trochanter and was compared with the planned value. The hip offset was measured preoperatively and postoperatively and the variation was compared with the planned offset change. The accuracy of the planned lengthening was evaluated measuring the change in the hip length compared with the preoperative state.

### 2.3. Surgical Time Evaluation

The surgical time was recorded for each procedure. The data regarding the length of surgery were compared with those collected in a matched group of 36 patients who underwent a conventional THA over the same time period. The patients were matched by age, BMI and ASA score. The matched group consisted of patients operated on by the same surgeon using the same implant system and the same surgical approach.

### 2.4. Statistical Analysis

The statistical analysis was conducted with STATA software 14.1. The variables were expressed as means (standard deviation). The normality of the data was verified by the Shapiro–Francia test. For the variables whose distribution was normal, a Student’s *t*-test for paired samples was performed while for the variables whose distribution was not normal, a Wilcoxon’s signed rank test was used. *p* < 0.05 was considered to be significant.

## 3. Results

All patients were available at follow-up; the final assessment included 36 patients (19 men and 17 women) with a mean age of 72 years (range of 58–79 years).

No complications such as an early infection or a dislocation were reported at a mean follow-up of 11.7 months (range 6–17 months).

The average planned and postoperative anteversion of the cup was 20.8° (17° to 25°) and 18.3° (5° to 30°), respectively, and the average planned and postoperative inclination of the cup was 39.4° (32° to 44°) and 38.4° (28° to 50°), respectively. The average level of the osteotomy was 10.26 mm (range 5–24 mm) in preoperative planning and 10 mm (range 4–26 mm) at the postoperative evaluation. The mean postoperative offset variation was 1 mm (−4 to 8 mm) and did not statistically differ from the mean planned offset change that was 2 mm (−2 to 5 mm). The effective hip length change compared with the preoperative status was 2.4 mm and did not significantly differ from the mean planned lengthening of 2.5 mm.

No statistically significant difference was detected comparing the planned and the achieved values (Table 1).

**Figure 7:**
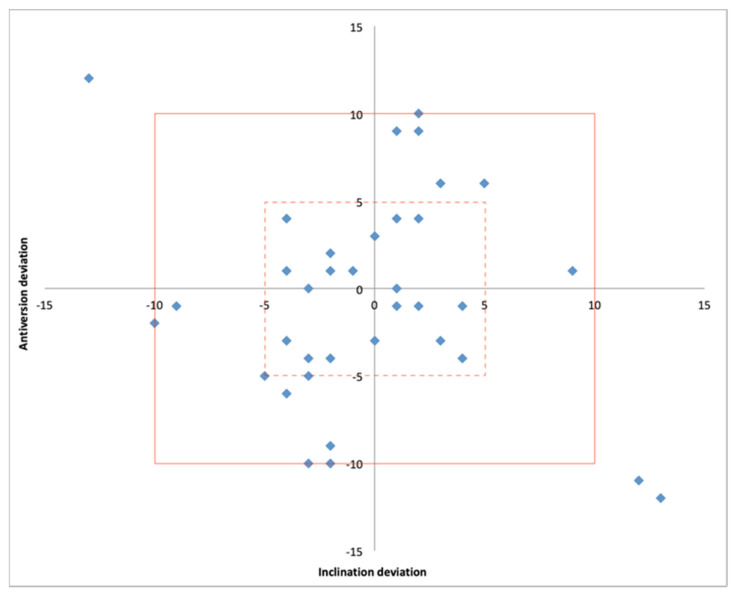
Scatter plot showing the position of the acetabular component within 5° (hashed line box) and 10° (solid line box) of deviation from the planned inclination and anteversion.

The size of the acetabular cup matched that in the preoperatively plan in 36/36 cases. The mean absolute deviation from the planned patient-specific inclination and anteversion was 3.9° (0.0° to 13°) and 4.4° (0.0° to 12°), respectively. Both the postoperative inclination and anteversion were within +/− 5° of the planned values in 58% of cases while a deviation within +/− 10° was detected in 92% of cases (Table 2) (Figure 7).

The femoral stem size matched what was preoperatively planned in 35/36 cases. The mean absolute deviation from the planned height of the resection was 1.6 mm (range 0–4 mm). In 75% of cases, the level of the osteotomy was found to be within 2 mm of the planned height of the resection. All osteotomies were within 4 mm of the planned level.

The deviation from the planned offset change was found to be within 4 mm (range 0–8 mm) in 83% of patients while in all cases it was within 8 mm.

The mean absolute deviation regarding the hip length change was 2 mm (range 0–5 mm); in 92% of patients, it was within 4 mm of the lengthening planned preoperatively.

The results are summarized in Table 3.

A statistically significant difference regarding the length of surgery was detected comparing the OPS^TM^ and standard technique (*p* = 0.0001). The mean surgical time was 71.4 (61 to 84) min in the OPS^TM^ group and 60.4 (46 to 74) min in the conventional THA group. The average time was 11 minutes longer using OPS^TM^ PSI guides and the laser-guided technique.

## 4. Discussion

The main findings of this study highlighted that the OPS^TM^ PSI guides and laser-guided technique accurately reproduced dynamic planning with regard to the size and orientation of components, osteotomy level and change in length and offset. This modern technique requires a relatively longer surgical time compared with conventional surgery; however, it can intraoperatively deliver a patient-specific functional placement of the implant.

The component positioning and dynamic function are being extensively studied as significant factors in THA stability and survival [9,19,23,24]. The current literature focuses on understanding individual lumbopelvic sagittal kinematics and the relationship with functional cup alignment during daily life activities [13,25,26,27].

A variation in the sagittal pelvic tilt can occur in supine, standing and sitting positions and has a substantial effect on the functional anteversion and inclination of the acetabulum [9,24,28].

As the pelvis rotates posteriorly, the functional anteversion of the acetabulum will increase by 0.7° for each degree of posterior PT [24]. This rotation will prevent posterior dislocation and edge loading in a flexion but it can lead to posterior impingement and anterior instability in an extension. As an anterior rotation occurs, the functional anteversion is reduced thus preventing an anterior dislocation and edge loading in an extension but not in a flexion [8,13,28].

Moreover, a wide inter-individual variation exists [9,29] with the arc of pelvic motion in several patients being as mobile as 70° and in others as stiff as 5° [8].

Furthermore, in a ‘stiff’ spine, as a result of lumbar degeneration, flatback deformity or spine fusion, two types of abnormal sagittal kinematics, can occur. Lumbopelvic stiffness can cause an insufficient pelvic retroversion when sitting and an increased pelvic retroversion when standing. This generates an unsafe functional acetabular orientation and might be responsible for a few complications such as impingement, edge loading and prosthetic instability. Such variations suggest that Lewinneck’s fixed safe zone may not fit for all patients; components may appear well-oriented on standard views or on the operative table but become poorly placed during more functionally-relevant postures [10,13,30]. As a result, the utility of the femoral and acetabular component safe zone has been questioned and, currently, there is an increasing paradigm shift from conventional techniques (mechanical alignment and combined anteversion techniques) toward techniques considering the functionality of the cup orientation. These latter techniques have been defined as lumbopelvic kinematics and spine-hip relation adjusted techniques (or kinematic alignment in a THA), with the aim of reaching a functional interaction of components and preventing poor interaction, edge loading and articular impingements from standing to sitting positions [19,30].

A dynamic hip positioning based on a more comprehensive radiological study evaluating lumbopelvic kinematics in supine, flexed-seated and standing positions has been reasonably proposed for a more physiological, functional and patient-specific adapted device [8,13,16].

While numerous studies on the cup position in a THA are available, there are limited data on the femoral stem position [18,31,32]. Recent literature has reported the importance of planning the femoral osteotomy for a successful THA as the femoral neck resection influences the leg length, offset and the tracking of the implant [17,33]. Errors in the leg length and offset can severely affect the function and the quality of life of the patients as well as prosthetic survival. Moreover, leg length discrepancy is one of the common reasons behind a patient’s complaining, dissatisfaction and litigation [34,35]. Stem anteversion and alignment in the frontal plane are often related to the shape of the proximal femur [36]. A few studies investigating the differences of the medullary canal at different levels of a neck resection [37,38] noticed how the actual morphology of the femoral canal varies significantly, resulting in possible differences in stem orientation [32]. As a result, the femoral offset is strongly affected by the femoral neck resection angle and stem positioning. Recreating an appropriate offset is essential for optimizing the abductor muscle strength, range of motion and stability [39,40].

The Optimized Positioning System^TM^ (Corin, Cirencester, UK) is a technological version of the KA (kinematic alignment) technique, which aims to reduce the risk of a poor functional component interaction. It aims to achieve an ideal cup position and to restore most of the hip anatomy through 3D planning and technological implantation assistance [16,41,42].

Dynamic planning has to be accurately reproduced in the operating room to finalize the functional advantages. The malposition of components mainly results from the errors in patient positioning, intraoperative pelvic motion and manual errors during the surgery [43]. Traditional free hand techniques successfully achieve target ranges for both inclination and anteversion in only 47% of cases with other studies reporting even lower rates [20,21,44].

Several techniques including the use of computer navigation, robotic surgery and PSI can improve those results and better achieve a planned target. It has been reported that 80% to 96% of components were placed in the safe zone using computer navigation while 100% were placed using robotics. In our series, all of the postoperative acetabular components were within Lewinnek’s safe zone [43].

Navigation and robotics, however, have a limited use because of high costs, increased surgical time and other logistical issues [45,46]. More recently, PSI guides have been shown to improve the accuracy of implant positioning in a THA compared with standard instrumentation and may play an important role in reconstructing complex anatomy. It has been reported that the use of PSI for acetabular components reduces the number of outliers compared with the use of standard instrumentation (0% versus 23.7%, respectively) [47].

Small et al. [48] compared 18 conventional THAs and 18 THAs with PSI, evaluating the planned versus the postoperative results using CT. The authors found a mean difference from the planned versus the postoperative cup anteversion of −6.9° for the standard instrumentation and −0.2° for the PSI cases. The difference for the inclination was not statistically significant.

Spencer-Gardner et al. [16] evaluated the OPS^TM^ PSI laser guidance system for the cup placement in 100 patients using postoperative 3D CT. They found that the postoperative inclination and anteversion were within +/− 5° of the planned values in 54% of cases while a deviation within +/− 10° was detected in 91% of cases. These results were similar to our findings and comparable with robotic and computer-navigated techniques.

In the study conducted by Schneider et al. [33], the OPS™ patient-specific femoral guide reproduced the planned osteotomy level within 3 mm in 29 out of 30 patients. The authors used posterolateral osteotomy guides with a minimally-invasive direct superior approach. In our series, all surgeries were performed with a direct lateral approach using an anterior femoral guide. However, comparable results were detected as in 75% of cases, the level of the osteotomy was within 2 mm of the planned height of the resection and in all patients, it was within 4 mm of the planned level.

In our study we evaluated both acetabular and femoral instrumentation. We also assessed offset and limb length change, which did not significantly differ from the planned estimated change.

The reliability of preoperative planning was also confirmed by the excellent match between the planned and actual size of the implanted components, with only one case of a mismatch in the stem size.

Regarding the surgical time, Spencer-Gardner et al. [16] reported an additional 3 to 5 minutes using OPS^TM^ acetabular patient-specific instrumentation while for the OPS^TM^ femoral guide, approximately an additional 3 min was reported by Schneider et al. [33]. In the present series, which included a learning curve, the operating time using both acetabular and femoral PSI increased by a mean of only 11 min (range of 8 to 14 min) when compared with a standard technique. The mean surgical time for a navigated THA has been reported to increase by up to 58 min [49].

It is important to point out that, in this study, the surgeon strictly followed the OPS^TM^ preoperative plan with the aid of PSI instruments to position the implants. In none of the cases did the surgeon change the reaming direction or the angle and level of femoral resection as is often needed in a PSI total knee arthroplasty [50].

It needs to be considered that a few conditions could limit the use of a dynamic THA in several patients. Severe bilateral hip arthrosis and stiffness could make it difficult to obtain reliable preoperative images as a proper flexed-seated position could barely be achieved and maintained.

Furthermore, the depth of the acetabular reaming is still determined by hand and the femoral PSI does not guide the stem version.

To the best of our knowledge, this is the first study aimed at evaluating simultaneously the accuracy of femoral and acetabular PSI along with the planned limb length and offset change in a kinematic THA using a direct lateral approach. Moreover, the surgical time was compared with a conventional THA.

This study has several potential limitations including the small number of patients and the absence of a clinical evaluation at an adequate follow-up. Nevertheless, follow-up is not a major concern as the primary outcome of the study was to assess the reliability of the PSI guides and laser-guided operative instrumentation to replicate preoperative dynamic planning and to determine the impact of the delivery system in the operating time.

However, the strength of this study is the accurate postoperative evaluation, performed with a CT scan, which allowed a three-dimensional and precise measurement of all values.

## 5. Conclusions

Based on the results of this study we can conclude that the delivery system of OPS^TM^ is safe and accurate in terms of cup and stem position and is reproducible and reliable. The increase in the operating time, which also included the learning curve, is negligible and did not limit the use of the technology. Further studies and a longer follow-up are needed to evaluate the clinical advantages of a dynamic THA as well as the cost-benefit ratio.

## Figures and Tables

**Table 1:** Mean planned and postoperative values.

	Planned Value (sd)	Postoperative Value (sd)	*p*-Value
Cup Inclination	39.4° (3.95)	38.4° (6.3)	0.3127
Cup Anteversion	20.8° (3.43)	18.3° (7.17)	0.0930
Osteotomy Height	10.3 mm (6.31)	10 mm (7.28)	0.4413
Offset Change	2 mm (2.31)	1 mm (3.3)	0.1678
Length Change	2.5 mm (1.86)	2.4 mm (3.09)	0.9305

Data are displayed as a mean and standard deviation.

**Table 2:** Deviation from the planned cup values.

	Absolute Deviation, Mean (Range)	% Within ± 5° (*n*)	% Within ± 10° (*n*)
Cup Inclination	3.9° (0–13)	83% (30)	92% (33)
Cup Anteversion	4.4° (0–12)	67% (24)	92% (33)
Both I and A		58% (21)	92% (33)

Data are displayed as a mean, number and percentage. I = inclination; A = anteversion.

**Table 3:** Deviation from the planned values.

	Absolute Deviation, Mean (Range)	% Within ± 2 mm (*n*)	% Within ± 4 mm (*n*)
Osteotomy Height	1.6 mm (0–4)	75% (27)	100% (36)
Offset Change	2.6 mm (0–8)	58% (21)	83% (30)
Length Change	2 mm (0–5)	67% (24)	92% (33)

Data are displayed as a mean, number and percentage.
